# PD65 Pilot Project Of Horizon Scanning To Inform The Ukrainian Health System About New HIV/AIDS Health Technologies

**DOI:** 10.1017/S0266462325101979

**Published:** 2025-12-29

**Authors:** Oresta Piniazhko, Elizaveta Otrubchak, Tamara Mahanova, Iryna Musiienko, Valeriia Serediuk, Iuliia Malyshevska, Iñaki Gutiérrez Ibarluzea, Mykhaylo Babenko, Mykhailo Lobas, Olena Filiniuk, Taras Lyaskovskyi, Rabia Sucu

## Abstract

**Introduction:**

Horizon scanning is an advanced health technology assessment (HTA) tool integrated into the decision-making process to proactively inform stakeholders about new health technologies. The objective of this pilot was to conduct horizon scanning for new health technologies for HIV/AIDS that could be appropriate for Ukraine’s health system, and to assess prospects for using the horizon scanning tool in Ukraine.

**Methods:**

The pilot project was conducted at the State Expert Centre of the Ministry of Health in Ukraine. The pilot project tested the basic horizon scanning methodology as well as the PRITECTOOLS prioritization tool using a dataset compiled from international and local databases of clinical trials, patents, academic publications, international horizon scanning and early assessment databases, media resources (including online resources), and websites of public and charitable organizations.

**Results:**

The team identified the following new medicines that may be relevant for Ukraine: cabotegravir (long-acting injectable integrase strand transfer inhibitor—an alternative to tablet-based regimens for daily pre-exposure prophylaxis to increase patient adherence); ibalizumab. an injectable CD4 receptor inhibitor that, in combination with other antiretroviral therapy, is indicated for treatment of HIV-1 in adults with multidrug-resistant HIV (more than five lines of therapy) to expand access and coverage of HIV treatment services; and dapivirine vaginal ring, a vaginal ring used to reduce the risk of HIV-1 infection in women during sexual intercourse to increase adherence to pre-exposure prophylaxis.

**Conclusions:**

The pilot showed that the horizon scanning tool can be used to inform the Ukrainian healthcare system about new medicines for prevention, diagnosis, and treatment of HIV/AIDS. The tool can also be one of the stages in the HTA process in Ukraine for healthcare decision-makers to proactively anticipate and plan for HTA for priority health conditions.

